# A Fermented Herbal Formulation Improves Intestinal Health and Growth Performance in Post-Weaning Piglets

**DOI:** 10.3390/ani16081254

**Published:** 2026-04-19

**Authors:** Xu Wang, Xin Fan, Chengying Li, Pinpin Chen, Shijie Li, Jintao Peng, Wei Zhou, Zutao Zhou, Xiaowen Li, Jiakui Li, Yuncai Xiao

**Affiliations:** 1 National Key Laboratory of Agricultural Microbiology, Huazhong Agricultural University, Wuhan 430070, China; wangxu1994@webmail.hzau.edu.cn (X.W.); fanxin1234@webmail.hzau.edu.cn (X.F.); wzcpp@webmail.hzau.edu.cn (P.C.); lishijie@webmail.hzau.edu.cn (S.L.); pjt@webmail.hzau.edu.cn (J.P.); zhouwei0715@mail.hzau.edu.cn (W.Z.); ztzhou@mail.hzau.edu.cn (Z.Z.); 2 College of Veterinary Medicine, Huazhong Agricultural University, Wuhan 430070, China; 3 Key Laboratory of Preventive Veterinary Medicine in Hubei Province, Huazhong Agricultural University, Wuhan 430070, China; 4 Eastern Along Pharmaceutical Co., Ltd., Foshan 528234, China; lichengying2008@163.com; 5 Hubei Huada Real Science & Technology Co., Ltd., Wuhan 432700, China; misscristine@163.com

**Keywords:** fermented herbal formulation, IPEC-J2 cells, weaned piglets, intestinal barrier function, inflammatory response, gut microbiota, short-chain fatty acids (SCFAs)

## Abstract

Weaning impairs intestinal function and growth performance in piglets. This study evaluated a fermented herbal formulation (FHF) composed of five bioactive herbal ingredients—*Radix isatidis*, *Folium isatidis*, *Radix scutellariae*, *Fructus forsythiae*, and *Radix glycyrrhizae*—fermented with *Enterococcus faecium* and *Saccharomyces cerevisiae* and characterized by flavonoids, phenolic acids, and hydroxylated fatty acids, using the porcine intestinal epithelial cell line (IPEC-J2) and weaned piglets. In vitro, IPEC-J2 cells were pretreated with FHF extract (100–1000 μg/mL) for 3 h prior to lipopolysaccharide (LPS) challenge. In vivo, 72 piglets were weaned at 32 days of age and, after a 3-day post-weaning adaptation period, entered a 35-day feeding trial. The piglets were then randomly assigned to three treatment groups: control (basal diet), A1 (basal diet + 0.4% FHF), and A2 (basal diet + 0.6% FHF during days 1–18, followed by 0.3% FHF during days 19–35). FHF dose-dependently alleviated the LPS-induced decrease in cell viability and suppressed IL-6, IL-8, IL-1β, and TNF-α expression. In piglets, the A2 group showed higher final body weight, average daily gain (ADG), and average daily feed intake (ADFI), lower feed conversion ratio (FCR), and a lower diarrhea rate than the control group. FHF also improved intestinal morphology, reduced serum TNF-α and diamine oxidase (DAO) levels, increased jejunal tight junction protein expression, enriched *Limosilactobacillus* and *Lactobacillus*, and elevated acetic and butyric acids. FHF improved intestinal health and growth performance in weaned piglets, with the A2 group showing the best overall efficacy.

## Simple Summary

Weaning is a major challenge in pig production because piglets often experience reduced feed intake, diarrhea, impaired intestinal function, and slower growth during this period. With the reduced use of in-feed antibiotics, effective nutritional alternatives have become increasingly important for supporting intestinal health. In this study, we evaluated a fermented herbal formulation (FHF) composed of *Radix isatidis*, *Folium isatidis*, *Radix scutellariae*, *Fructus forsythiae*, and *Radix glycyrrhizae*, and fermented with *Enterococcus faecium* and *Saccharomyces cerevisiae*. Piglets were weaned at 32 days of age and entered the feeding trial after a 3-day post-weaning adaptation period. FHF protected porcine intestinal epithelial cells against inflammatory injury and improved growth performance, reduced diarrhea, and enhanced intestinal morphology in weaned piglets. It also increased the expression of key proteins involved in maintaining the intestinal barrier. In addition, FHF shifted the gut microbial community toward more beneficial bacteria and increased short-chain fatty acids, especially acetate and butyrate. Among the tested regimens, the step-down supplementation strategy gave the best overall results. These findings support the potential use of FHF as a functional feed additive for improving intestinal health during the post-weaning period in piglets.

## 1. Introduction

Under natural conditions, piglets are usually weaned at 12–17 weeks of age, whereas commercial production systems typically wean them at 3–4 weeks, depending on management practices [1]. The post-weaning period is a critical phase in swine production, during which piglets are exposed to nutritional, environmental, and psychological stressors, frequently leading to intestinal inflammation, barrier dysfunction, and gut microbiota dysbiosis [2,3]. These disorders can contribute to increased diarrhea incidence and poor growth performance, causing substantial economic losses [4]. With stricter regulations on in-feed antibiotics due to concerns over antimicrobial resistance and drug residues, there is an increasing trend within the swine industry to explore safe and effective alternative dietary additives [5]. Traditional Chinese herbal mixtures (CHMs) are abundant in various bioactive compounds, which present significant potential for promoting animal health [6,7]. For example, *Radix isatidis* and *Folium isatidis* exhibit antiviral and anti-inflammatory activities [8], while *Radix scutellariae* is rich in flavonoids with antioxidant and immunomodulatory effects [9]. Additionally, *Fructus forsythiae* exhibits broad-spectrum antimicrobial properties [10], and *Radix glycyrrhizae* can protect the intestinal mucosa [11]. However, the direct use of raw herbal materials in animal diets is often limited by poor palatability, low bioavailability of active compounds, and inconsistent efficacy [12]. Accordingly, microbial fermentation has been proposed as an effective approach to improve their utilization by promoting biotransformation of bioactive compounds and generating beneficial metabolites, such as organic acids and bioactive peptides [13,14].

In recent years, fermented CHMs have received growing attention as functional feed additives. Fermentation with probiotic strains, such as *Lactobacillus* spp. and *Saccharomyces cerevisiae*, not only improves the biological activities of herbal constituents but also promotes a healthier gut ecology [13]. Research indicates that the inclusion of fermented CHMs in piglet diets can yield significant improvements. For example, dietary supplementation with a fermented herbal mixture (Zhihuasi Tk) consisting of *Codonopsis pilosula* and *Astragalus membranaceus* was shown to improve average daily gain and decrease the diarrhea rate in weaned piglets [15]. Furthermore, fermented Chinese herbal residues improved colonic health by increasing short-chain fatty acid concentrations and regulating genes related to barrier function [16]. In addition, a fermented multi-herb formulation containing *Atractylodes macrocephala*, *Portulaca oleracea*, *Astragalus*, and other herbs was reported to improve villus morphology and nutrient digestibility in weaned piglets [17]. These findings suggest that fermented CHMs could facilitate animal growth and maintain intestinal health during the weaning transition.

FHF is a fermented herbal formulation composed of five traditional Chinese herbal ingredients—*Radix isatidis*, *Folium isatidis*, *Radix scutellariae*, *Fructus forsythiae*, and *Radix glycyrrhizae*—selected for their anti-inflammatory, antioxidant, and mucosal-protective activities. Although FHF has shown beneficial effects in reducing diarrhea and improving overall health in clinical veterinary practice, little information is available regarding its specific effects on the intestinal homeostasis of weaned piglets. Therefore, we hypothesized that dietary FHF supplementation could help alleviate post-weaning intestinal dysfunction by suppressing inflammation, enhancing barrier integrity, and modulating the composition of intestinal microbiota. The objective of this study was to evaluate the effects of FHF on intestinal barrier function, inflammatory responses, and growth performance using both an LPS-challenged IPEC-J2 cell model and a weaned piglet feeding trial. This work provides experimental evidence supporting the application of fermented herbal formulations as functional feed additives to improve intestinal health in piglets.

## 2. Materials and Methods

### 2.1. Extraction and Chemical Analysis of FHF

FHF was provided by Hubei Huada Real Science & Technology Co., Ltd. (Wuhan, China). The chemical composition of the FHF is shown in Table 1.

A total of 10 g of FHF powder was decocted in 200 mL of ultrapure water for 2 h until the volume was reduced to 100 mL. The decoction was centrifuged at 8000× *g* for 15 min at 4 °C and filtered sequentially through sterile gauze and a 0.22 μm membrane filter (Merck Millipore, Burlington, MA, USA). The sterilized extract was stored at −80 °C. The preparation procedure was based on previous protocols for herbal extraction [15].

Chemical characterization of the FHF extract is described in Supplementary Materials and Methods S1.1. The classification of identified metabolites is presented in Supplementary Figure S1, and the top 20 bioactive compounds are provided in Supplementary Table S1.

### 2.2. Cell Treatment

IPEC-J2 cells were provided by the College of Veterinary Medicine, Huazhong Agricultural University, Wuhan, China.IPEC-J2 is a non-transformed porcine jejunal epithelial cell line derived from neonatal piglet jejunum and is widely used for in vitro studies of intestinal barrier function and epithelial inflammatory responses. Cell culture was carried out in DMEM (Gibco, Grand Island, NY, USA) supplemented with 10% fetal bovine serum (Gibco, Grand Island, NY, USA) and 1% penicillin–streptomycin (Gibco, Grand Island, NY, USA) at 37 °C in 5% CO_2_. Cell viability was assessed with a CCK-8 kit (Serana, Shanghai, China) following the supplier’s protocol. For the viability assay, cells were plated in 96-well plates at 1 × 10^4^ cells per well. After the indicated treatments, 10% CCK-8 reagent was added and the plates were incubated for 2 h, after which absorbance was recorded at 450 nm. For cytoprotection experiments, cells at 80–90% confluence were randomly assigned to five groups: (1) Control group: serum-free DMEM for 15 h; (2) LPS group: serum-free DMEM for 3 h followed by 10 μg/mL LPS (Sigma-Aldrich, St. Louis, MO, USA; Cat. No. L2880) for 12 h; (3–5) FHF + LPS groups: pretreated with FHF extract (100, 500, or 1000 μg/mL) for 3 h, followed by 10 μg/mL LPS challenge for 12 h.

### 2.3. Animal Trial Design

The animal trial was approved under protocol No. HZAUSW-2024-0006 at Huazhong Agricultural University. Seventy-two crossbred piglets (Duroc × Landrace × Yorkshire) were used in this study. They were weaned at 32 days of age. During the 3-day post-weaning adaptation period, all piglets were housed in the nursery facility and fed the same basal weaning diet, with ad libitum access to feed and water. After a 3-day post-weaning adaptation period, they entered the 35-day feeding trial with an average initial body weight of 9.41 ± 0.29 kg. This initial body weight was recorded at the start of the feeding trial, after the 3-day post-weaning adaptation period, rather than on the day of weaning, and therefore reflects the relatively later weaning age and post-adaptation measurement time. The piglets were assigned to three treatments with three pens per treatment and eight piglets in each pen, with males and females equally represented in each pen: (1) Control: basal diet; (2) A1: basal diet + 0.4% (4 kg/ton) FHF; and (3) A2: basal diet + 0.6% (6 kg/ton) FHF during days 1–18, followed by 0.3% (3 kg/ton) during days 19–35. The FHF was provided in powdered form, passed through a 60-mesh sieve, and mixed with the basal diet before feeding. The A2 group was designed as an exploratory step-down strategy because intestinal stress is more severe during the early post-weaning period. Therefore, a higher inclusion level was used in the initial phase, followed by a lower inclusion level in the later phase to sustain the effect. All piglets had ad libitum access to feed and water throughout the experiment. A farm-formulated basal weaning diet was used, and its ingredient and nutrient composition is shown in Supplementary Table S2. Room temperature was set at 28 ± 1 °C for days 1–14 and 26 ± 1 °C for days 15–35, with relative humidity maintained at 50–60% under routine nursery conditions and a 12 h light/dark cycle. Growth and feed-use indices were calculated from body-weight records together with daily feed intake data.

### 2.4. Sample Collection

After completion of the 35-day trial, two piglets were chosen from each pen for sample collection (n = 6 per group). Following a 12 h fast, blood was taken from the anterior vena cava and serum was separated by centrifugation at 3000× *g* for 10 min. Piglets were then sacrificed with sodium pentobarbital administered intravenously (30 mg/kg body weight), followed by exsanguination. Samples of the spleen, duodenum, jejunum, ileum, and colonic digesta were collected and immediately preserved in liquid nitrogen before storage at −80 °C. Additional intestinal tissue was fixed in 4% paraformaldehyde for histological analysis.

### 2.5. Histological and Immunofluorescence Analyses

Histological evaluation and immunofluorescence staining of intestinal tissues were performed as described in Supplementary Materials and Methods S1.2.

### 2.6. ELISA Analysis

ELISA was used to quantify inflammatory cytokines in IPEC-J2 culture supernatants and pig serum with commercial kits from Meimian (Yancheng, China). In the cell experiment, supernatants were prepared after treatment and kept at −80 °C before measurement. IL-6 (Cat. No. MM-041802), IL-8 (Cat. No. MM-03705702), IL-1β (Cat. No. MM-042201), and TNF-α (Cat. No. MM-038301) were assayed in duplicate. In the animal experiment, serum samples were prepared and stored at −80 °C until use. The same four cytokines, together with diamine oxidase (DAO; Cat. No. MM-043801), were measured in duplicate. Absorbance was read at 450 nm, and concentrations were calculated from the corresponding standard curves.

### 2.7. Gene Expression Analysis

Gene expression was analyzed by quantitative real-time PCR (qRT-PCR) as described in Supplementary Materials and Methods S1.3. The primer sequences are listed in Supplementary Table S3.

### 2.8. Protein Expression Analysis

Protein expression was examined by Western blot as described in Supplementary Materials and Methods S1.4.

### 2.9. 16S rRNA Gene Sequencing

Microbial DNA was prepared from colonic contents with a QIAamp Fast DNA Stool Mini Kit (QIAGEN, Hilden, Germany). DNA quality was assessed by NanoDrop ND-2000 (Thermo Fisher Scientific, Waltham, MA, USA) measurement together with 1% agarose gel electrophoresis. The V3–V4 region of bacterial 16S rRNA was amplified with primers 341F (5′-CCTAYGGGRBGCASCAG-3′) and 806R (5′-GGACTACNNGGGTATCTAAT-3′) using a high-fidelity polymerase (Roche, Basel, Switzerland). The amplicons were cleaned with AMPure XP beads (Beckman Coulter, Brea, CA, USA), quantified with a Qubit 4.0 fluorometer (Thermo Fisher Scientific, Waltham, MA, USA), and then sequenced on an Illumina NovaSeq 6000 platform (Illumina, San Diego, CA, USA). Sequence processing was carried out in QIIME 2 (version 2023.2). After removal of low-quality and chimeric sequences, amplicon sequence variants (ASVs) were generated with DADA2, and taxonomic annotation was performed against the SILVA 138.1 database.

### 2.10. Short-Chain Fatty Acids (SCFAs) Analysis

SCFAs in colonic contents were quantified by gas chromatography–mass spectrometry (GC–MS system, Agilent Technologies, Santa Clara, CA, USA). Briefly, frozen samples were homogenized in distilled water, acidified with metaphosphoric acid containing 2-ethylbutyric acid as an internal standard, centrifuged, and filtered before analysis. SCFA concentrations were calculated from calibration curves prepared with analytical-grade standards. Detailed instrumental conditions are provided in Supplementary Materials and Methods S1.5.

### 2.11. Statistical Analysis

Statistical analyses were carried out in GraphPad Prism 9.5.1 (GraphPad Software, San Diego, CA, USA). Data are presented as mean ± standard error of the mean (SEM). Comparisons among multiple groups were performed by one-way analysis of variance (ANOVA) followed by Tukey’s post hoc test. Correlation analysis was performed using Spearman correlation coefficient. A value of *p* < 0.05 was taken as statistically significant, whereas 0.05 ≤ *p* < 0.10 was interpreted as a trend. In the figures, statistical significance is indicated as *p* < 0.05 (*), *p* < 0.01 (**), and *p* < 0.001 (***).

## 3. Results

### 3.1. Cytocompatibility and Cytoprotective Effects of FHF

**Figure 1:**
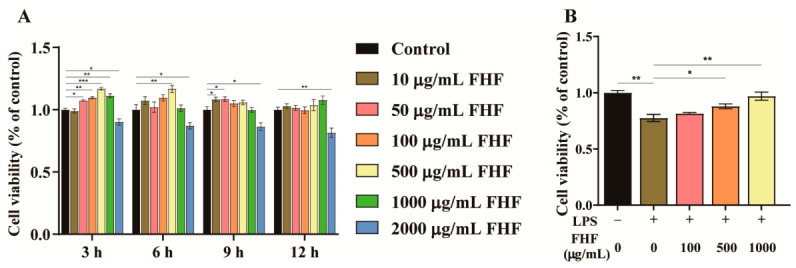
Effects of FHF extract on viability of IPEC-J2 cells. (**A**) Cell viability after treatment with 0–2000 μg/mL FHF for 3–12 h. (**B**) Cell viability after 3 h pretreatment with 100–1000 μg/mL FHF followed by 10 μg/mL LPS exposure for 12 h. *p* < 0.05 (*), *p* < 0.01 (**), *p* < 0.001 (***).

After 3 h of exposure, concentrations exceeding 50 μg/mL induced a biphasic response in cell viability, which was initially enhanced and then declined (Figure 1A). The concentration range that elicited this response progressively narrowed as the exposure duration was extended. Notably, the highest concentration tested (2000 μg/mL) consistently suppressed cell viability at all four time points (Figure 1A). Based on these results, a concentration range of 100–1000 μg/mL and an exposure time of 3 h were selected for all subsequent experiments.

To evaluate the protective efficacy of FHF, IPEC-J2 cells were stimulated with 10 μg/mL LPS, which significantly reduced cell viability to 77.45 ± 3.21% compared to the untreated control (*p* < 0.05; Figure 1B), and this reduction was dose-dependently attenuated by pretreatment with FHF extract (100, 500, and 1000 μg/mL) for 3 h prior to LPS challenge. The highest concentration of FHF (1000 μg/mL) restored cell viability to 97.12 ± 3.68%, which was close to the untreated control level and higher than that observed in the LPS group (*p* < 0.01).

### 3.2. Dose-Dependent Suppression of LPS-Induced Cytokines by FHF Extract

**Figure 2:**
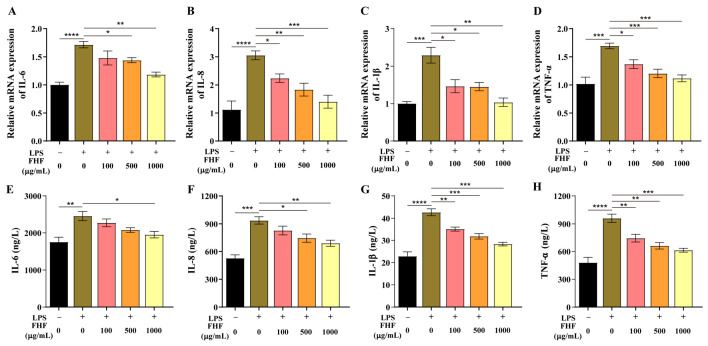
Inhibitory effect of FHF on LPS-induced cytokine expression in IPEC-J2 cells. (**A**–**D**) Relative mRNA levels of *IL-6*, *IL-8*, *IL-1β*, and *TNF-α*. (**E**–**H**) Corresponding protein levels of IL-6, IL-8, IL-1β, and TNF-α. *p* < 0.05 (*), *p* < 0.01 (**), *p* < 0.001 (***), *p* < 0.0001 (****).

LPS exposure markedly increased the mRNA levels of the pro-inflammatory cytokines *IL-6*, *IL-8*, *IL-1β*, and *TNF-α* (*p* < 0.001; Figure 2A–D). This response was reduced in a dose-dependent manner by FHF pretreatment (100, 500, and 1000 μg/mL) for 3 h before LPS challenge. Suppression of all four cytokine transcripts was evident at 500 and 1000 μg/mL (*p* < 0.05; Figure 2A–D). LPS also increased cytokine secretion, as reflected by higher protein levels of IL-6, IL-8, IL-1β, and TNF-α (*p* < 0.01; Figure 2E–H). Pretreatment with FHF lowered cytokine release in a dose-dependent manner and markedly reversed the increase in IL-1β and TNF-α across the tested concentrations (*p* < 0.01; Figure 2G,H). At 1000 μg/mL, all four cytokines were significantly reduced at the protein level (*p* < 0.05; Figure 2E–H).

### 3.3. FHF Supplementation Enhances Growth Performance and Reduces Diarrhea Incidence

**Figure 3:**
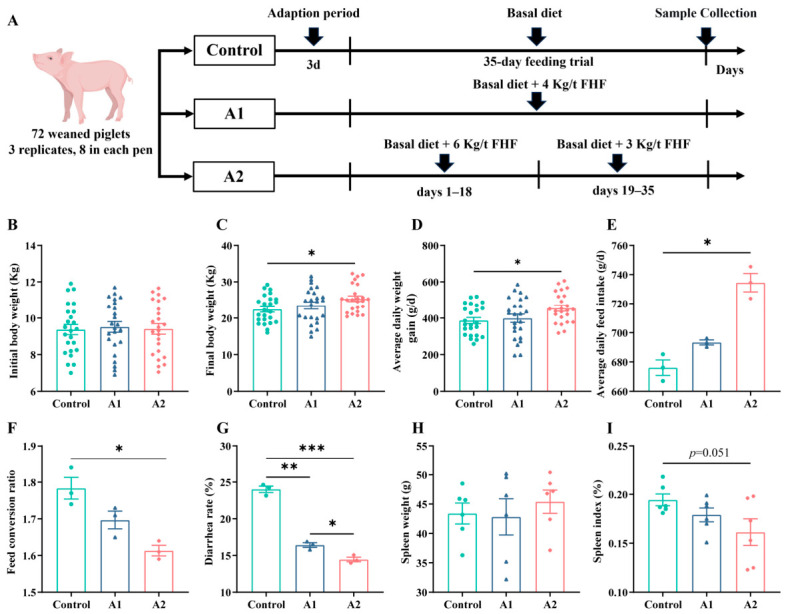
Growth performance, diarrhea rate, and spleen-related indices in weaned piglets receiving dietary FHF. (**A**) Schematic diagram of the experimental design. (**B**) Body weight at the start of the trial. (**C**) Final body weight. (**D**) Average daily gain (ADG). (**E**) Average daily feed intake (ADFI). (**F**) Feed conversion ratio (FCR). (**G**) Diarrhea rate. (**H**) Spleen weight. (**I**) Spleen index. Data are expressed as mean ± SEM. For (**B**–**D**), n = 24 pigs per group; for (**E**–**G**), n = 3 pens per group; and for (**H**,**I**), n = 6 pigs per group. *p* < 0.05 (*), *p* < 0.01 (**), *p* < 0.001 (***). A tendency was considered at 0.05 ≤ *p* < 0.10.

The study design is presented in Figure 3A. Initial body weights were similar among groups (Figure 3B). At day 35, piglets in the variable-dose A2 group showed significantly greater final body weight and ADG than controls (*p* < 0.05; Figure 3C,D). The A2 group also exhibited higher ADFI and a lower FCR (*p* < 0.05; Figure 3E,F), indicating improved nutrient utilization efficiency. Both FHF treatments markedly reduced diarrhea rate (*p* < 0.05; Figure 3G), reflecting enhanced intestinal homeostasis. Because the spleen is an important peripheral immune organ and is commonly used as an auxiliary indicator of systemic inflammatory status, it was also evaluated at necropsy. While absolute spleen weight did not differ (Figure 3H), the A2 group showed a tendency toward a lower spleen index (0.05 ≤ *p* < 0.10; Figure 3I), suggesting reduced systemic inflammatory stress.

### 3.4. Effects of FHF on the Intestinal Morphology in Piglets

Dietary supplementation with FHF significantly improved intestinal morphology of weaned piglets, although the effects varied across the different intestinal segments (Table 2). Representative H&E images of the small intestine are provided in Supplementary Figure S2. In the duodenum, both A1 and A2 showed higher VH (*p* < 0.001) together with an increased VH/CD ratio (*p* < 0.001), whereas CD remained unchanged among groups. In the jejunum, VH and CD were similar across treatments, but the VH/CD ratio was higher in both FHF-treated groups than in the control group (*p* < 0.01), indicating a more balanced mucosal turnover. In the ileum, VH was increased in A2 relative to the control group (*p* < 0.05). No group effect was detected for ileal CD or VH/CD ratio.

### 3.5. Effects of FHF on Inflammatory Cytokines in Weaned Piglets

**Figure 4:**
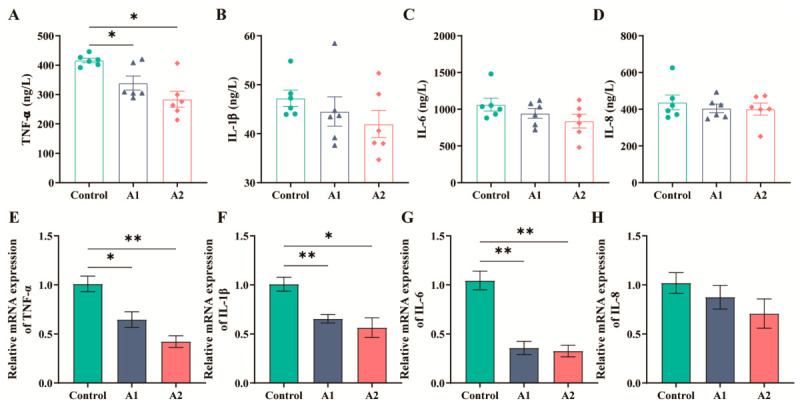
Serum cytokines and jejunal mucosal cytokine mRNA levels in weaned piglets receiving FHF. (**A**–**D**) Serum TNF-α, IL-1β, IL-6, and IL-8. (**E**–**H**) Jejunal mucosal mRNA levels of *TNF-α*, *IL-1β*, *IL-6*, and *IL-8*. Data are expressed as mean ± SEM (n = 6). *p* < 0.05 (*), *p* < 0.01 (**).

Serum TNF-α was lower in both FHF-treated groups than in the control group (*p* < 0.05), whereas no group differences were detected for serum IL-1β, IL-6, or IL-8 (Figure 4A–D). In the jejunal mucosa, *TNF-α*, *IL-1β*, and *IL-6* mRNA levels were reduced in A1 and A2 relative to the control group (*p* < 0.05), while IL-8 mRNA remained unaffected (Figure 4E–H). Representative immunofluorescence images (Supplementary Figure S3A) and quantitative analysis of mean fluorescence intensity (MFI; Supplementary Figure S3B) demonstrated that phosphorylated NF-κB p65 (p-p65) levels were significantly reduced in the A1 and A2 groups compared with the control group.

### 3.6. Effects of FHF on Intestinal Barrier Function in Weaned Piglets

**Figure 5:**
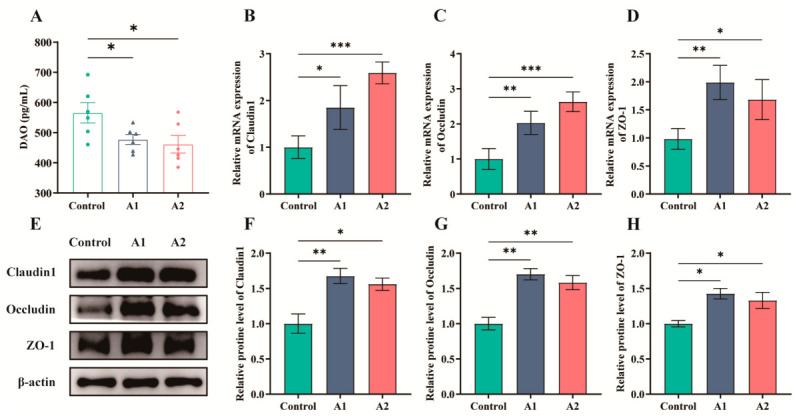
Intestinal barrier-related indices in weaned piglets receiving dietary FHF. (**A**) Serum levels of diamine oxidase (DAO). (**B**–**D**) Jejunal mRNA levels of *Claudin1*, *Occludin*, and *ZO-1*. (**E**) Representative Western blot bands of Claudin1, Occludin, and ZO-1. (**F**–**H**) Densitometric quantification of Claudin1, Occludin, and ZO-1 protein levels, respectively. *p* < 0.05 (*), *p* < 0.01 (**), *p* < 0.001 (***).

Serum DAO (diamine oxidase) was decreased after FHF supplementation (*p* < 0.05; Figure 5A). In the jejunum, FHF increased the mRNA abundance of the tight-junction genes *Claudin1*, *Occludin*, and *ZO-1* (*p* < 0.05; Figure 5B–D). Western blot analysis showed the same overall pattern, with increased protein abundance of Claudin1, Occludin, and ZO-1 in A1 and A2 (*p* < 0.05; Figure 5E–H).

### 3.7. Effects of FHF on Gut Microbiota Composition in Weaned Piglets

**Figure 6:**
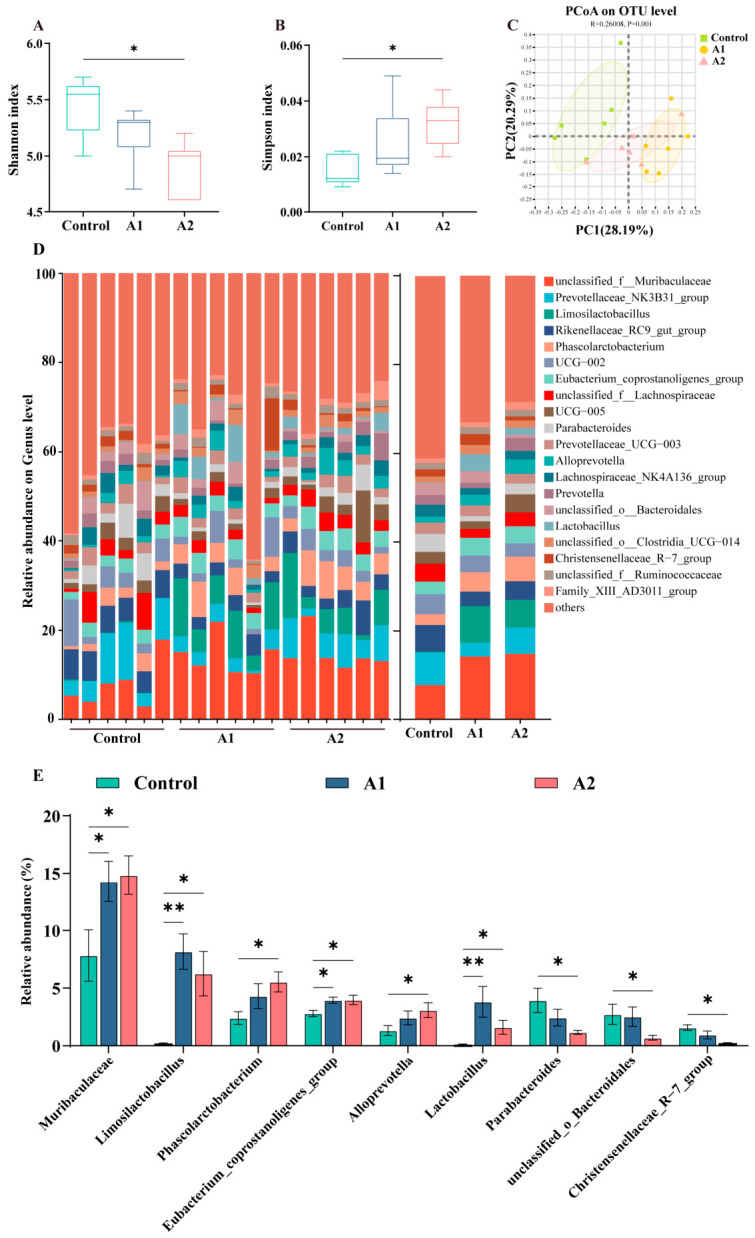
Colonic microbiota profiles in weaned piglets receiving dietary FHF. (**A**) Shannon index. (**B**) Simpson index. (**C**) Principal coordinate analysis based on Bray–Curtis dissimilarity. (**D**) Relative abundance of the top 20 genera in each sample. (**E**) Differentially abundant genera among the three groups. Data are expressed as mean ± SEM (n = 6). *p* < 0.05 (*), *p* < 0.01 (**).

Alpha diversity analysis indicated that the A2 group had a lower Shannon index but a higher Simpson index than the control group (*p* < 0.05; Figure 6A,B). Principal coordinate analysis based on Bray–Curtis dissimilarity revealed clear separation among the control, A1, and A2 groups (*p* < 0.05; Figure 6C). The distribution of the 20 most abundant genera is shown in Figure 6D. Genus-level analysis further showed enrichment of *Muribaculaceae*, *Limosilactobacillus*, *Lactobacillus*, and *Eubacterium_coprostanoligenes_group* after FHF supplementation (*p* < 0.05; Figure 6D,E). The A2 group further enriched *Phascolarctobacterium* and *Alloprevotella* while reducing *Parabacteroides*, *unclassified_o_Bacteroidales*, and *Christensenellaceae_R-7_group* (*p* < 0.05).

### 3.8. Effects of FHF on SCFA Production and Microbe–Metabolite Correlations

**Figure 7:**
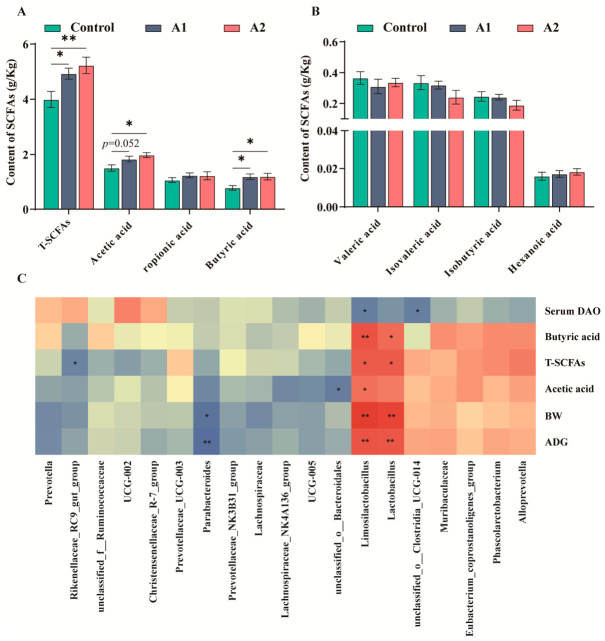
Effects of dietary FHF supplementation on colonic SCFA concentrations and microbe–metabolite correlations. (**A**) Concentrations of T-SCFAs, acetic acid, propionic acid, and butyric acid. (**B**) Concentrations of valeric acid, isovaleric acid, isobutyric acid, and hexanoic acid. (**C**) Spearman correlation heatmap between differentially abundant genera and selected parameters. Values are presented as mean ± SEM (n = 6). *p* < 0.05 (*), *p* < 0.01 (**). A tendency was considered at 0.05 ≤ *p* < 0.10.

Both FHF-treated groups showed increased concentrations of total SCFAs (T-SCFAs) and butyric acid (*p* < 0.05; Figure 7A). Acetic acid was also elevated in A2 (*p* < 0.05), whereas A1 showed a tendency toward a higher acetic acid concentration (0.05 ≤ *p* < 0.10; Figure 7A). No group effect was detected for propionic acid, valeric acid, isovaleric acid, isobutyric acid, or hexanoic acid (Figure 7B). Spearman correlation analysis showed that *Limosilactobacillus* and *Lactobacillus* were positively correlated with butyric acid, T-SCFAs, final body weight, and ADG, and *Limosilactobacillus* was also positively correlated with acetic acid (*p* < 0.05; Figure 7C). Conversely, *Parabacteroides* was negatively correlated with final body weight and ADG, while *Limosilactobacillus* was inversely associated with serum DAO (*p* < 0.05; Figure 7C).

## 4. Discussion

### 4.1. Cytoprotective and Anti-Inflammatory Effects of FHF in IPEC-J2 Cells

Cell viability assays revealed that FHF extract showed no cytotoxicity at 10–1000 μg/mL in IPEC-J2 cells after 3 h of exposure. Based on these results, three concentrations (100, 500, and 1000 μg/mL) were selected for subsequent experiments. Pretreatment with FHF reversed the LPS-induced decline in cell viability in a dose-dependent manner, and suppressed pro-inflammatory cytokines. These findings suggest that FHF has cytoprotective and anti-inflammatory effects on intestinal epithelial cells. These effects are likely attributable to the diverse bioactive compounds present in the FHF extract. UHPLC-MS/MS analysis of FHF identified various metabolites, with phenylpropanoids and polyketides as the dominant superclass (25.07%), followed by lipids and lipid-like molecules (15.73%) and alkaloids (8.05%) (Supplementary Figure S1). Among the top 20 most abundant compounds, hydroxylated fatty acids accounted for the majority, while ferulic acid, shikimic acid, and thymol were also identified as major bioactive constituents (Supplementary Table S1). Previous studies have indicated that these compounds possess anti-inflammatory properties. For instance, ferulic acid can alleviate intestinal inflammation and modulate inflammatory signaling pathways [18,19]. Furthermore, thymol was reported to suppress pro-inflammatory cytokine production in porcine intestinal epithelial cells through modulation of inflammatory signaling pathways [20,21]. In addition, hydroxylated fatty acids, for example, 13-hydroxy-9,11-octadecadienoic acid (13-HODE), have been reported to participate in intestinal inflammatory regulation [22,23]. The presence of these diverse anti-inflammatory compounds suggests that FHF can protect IPEC-J2 cells, providing experimental evidence for its further evaluation in vivo. However, the present study does not allow definitive identification of the major anti-inflammatory component in FHF. Further studies are needed to isolate and validate the key bioactive compounds and to clarify their individual and synergistic contributions to the anti-inflammatory effects of FHF.

### 4.2. Effects of FHF on Growth Performance and Diarrhea Rate

Earlier work has shown that microbial fermentation can significantly enhance the biological activity of Chinese herbal materials, improving growth performance and health in livestock [24,25]. For instance, fermented herbal mixtures have been shown to increase average daily gain and modulate gut microbiota in weaned piglets [26], and to increase serum immunoglobulin levels and antioxidant capacity in lactating sows [27]. In the present study, the A2 group achieved higher final body weight, ADG, and ADFI, while maintaining a lower FCR (*p* < 0.05). Additionally, both FHF groups significantly reduced diarrhea rate in piglets. Similar findings were noted by Zhao et al. [28], who found that fermented herbal materials reduced post-weaning diarrhea and improved nutrient digestibility in piglets. The better performance of the A2 group is probably associated with its higher initial dose during the early post-weaning stage, when piglets are particularly vulnerable to intestinal stress [29,30]. A similar phased feeding approach has been reported for spray-dried plasma in nursery pigs, in which the dietary inclusion level was reduced by 50% from phase 1 to phase 2, which is consistent with the concept of a higher level of supplementation during the early post-weaning period [31]. Furthermore, early nutritional interventions may help shape beneficial gut microbiota and support intestinal development [32]. The higher initial dose may have offered greater protection against intestinal inflammation and barrier disruption during the acute post-weaning period, whereas the reduced dose used later appeared sufficient to sustain the beneficial effects. From a production perspective, the improvements in ADG and FCR, together with the lower diarrhea rate, indicate potential practical benefits of FHF supplementation during the post-weaning stage. Since early post-weaning health is important for subsequent growth, these benefits may also support later production performance. However, the present study did not include a formal economic evaluation or assess whether these effects persist into the finishing stage, and this should be clarified in future studies.

### 4.3. Effects of FHF on Intestinal Barrier Function and Inflammatory Responses

The small intestine is central to nutrient uptake and therefore closely linked to growth performance in piglets [33]. VH, CD, and VH/CD are widely used histological indicators of intestinal function, with increased VH and VH/CD generally reflecting improved absorptive capacity and mucosal maturity [34]. Previous studies have shown that a fermented multi-herb formulation containing *Atractylodes macrocephala*, *Portulaca oleracea*, *Astragalus*, and other herbs improved villus morphology and nutrient digestibility in weaned piglets [17]. Similarly, our investigation revealed that FHF supplementation significantly increased VH in the duodenum and ileum, and improved the VH/CD ratio in the duodenum and jejunum (Table 2 and Supplementary Figure S2). These structural improvements likely contributed to the enhanced growth performance observed.

Intestinal barrier integrity depends largely on tight junction proteins, including Claudin1, Occludin, and ZO-1, which regulate paracellular permeability and prevent pathogen translocation [35,36]. In the present study, FHF supplementation upregulated the expression of these proteins in the jejunum. The jejunum was selected because it is a major site of nutrient absorption and is commonly used to evaluate intestinal barrier function in weaned piglets. Similar effects have been reported for fermented traditional Chinese medicine residues, which enhanced ZO-1 and Occludin expression at both mRNA and protein levels in weaned piglets [37]. Serum DAO is commonly regarded as an indicator of impaired intestinal barrier integrity and increased mucosal permeability [38]. Accordingly, the decreased serum DAO levels in the present study further supported the improvement of intestinal barrier function. These effects may be linked to fermentation-derived short-chain fatty acids that support epithelial renewal and help maintain tight junction integrity [39].

Weaning stress is often associated with excessive inflammatory responses that impair intestinal integrity and growth performance [3]. NF-κB signaling plays a central role in this inflammatory response; after activation, p65 translocates to the nucleus and promotes pro-inflammatory responses [40]. In this study, FHF supplementation significantly reduced serum TNF-α levels and downregulated jejunal cytokine mRNA expression. Additionally, the decreased phosphorylated NF-κB p65 fluorescence in the FHF groups suggests the inhibition of NF-κB activation. These findings align with our in vitro results, where FHF extract suppressed LPS-induced cytokine secretion in IPEC-J2 cells in a dose-dependent manner. Previous studies have also demonstrated that fermented plant extracts can significantly inhibit NF-κB activation through TLR4-independent pathways, while simultaneously reducing TNF-α levels [41]. The anti-inflammatory effects of FHF are probably associated with bioactive constituents such as flavonoids and organic acids. By attenuating inflammation, FHF may preserve nutrients and energy for growth and tissue repair [29].

### 4.4. Effects of FHF on Gut Microbiota Composition and SCFA Production

The gut microbiota plays a key role in maintaining intestinal homeostasis, particularly during the weaning period when microbial communities are highly susceptible to dietary interventions [42]. Previous studies have shown that fermented feed additives can modulate gut microbial composition by introducing beneficial microorganisms and fermentation-derived metabolites, thereby improving nutrient utilization and intestinal function [43,44]. In this study, beta diversity analysis revealed distinct clustering among the control, A1, and A2 groups, suggesting that FHF supplementation reshaped the microbial community structure. Although the Shannon index decreased in the A2 group, this decrease may reflect the selective enrichment of functional bacteria. These findings indicate that FHF effectively changed the gut microbiota during the critical post-weaning stage.

FHF supplementation enriched beneficial genera, including *Limosilactobacillus* and *Lactobacillus*, which are widely recognized for their probiotic roles in supporting mucosal homeostasis and barrier integrity [13,45]. The A2 group further increased *Phascolarctobacterium* and *Alloprevotella*, genera that have been reported to be associated with SCFA production [46]. SCFAs, particularly butyrate and acetate, are key microbial metabolites that support epithelial energy metabolism and suppress inflammatory signaling pathways such as NF-κB [47,48]. In our study, FHF supplementation significantly increased total SCFAs and butyric acid levels, with the A2 group also showing an elevated acetic acid concentration. To further examine the relationship between microbial changes and host responses, correlation analysis was performed using the major microbial genera and the representative indicators that showed treatment-related differences among groups. Correlation analysis further demonstrated that *Limosilactobacillus* and *Lactobacillus* were positively associated with butyric acid levels, final body weight, and ADG, while negatively correlated with serum DAO. These findings suggest that FHF promotes growth performance and barrier integrity by regulating SCFA-producing bacteria and enhancing microbial metabolite production. Taken together, FHF appears to establish a beneficial microbiota–metabolite axis that contributes to intestinal health and improved growth during the weaning transition.

## 5. Conclusions

Our study showed that dietary supplementation with the fermented herbal formulation FHF, particularly under the step-down regimen (0.6% during days 1–18 followed by 0.3% during days 19–35), significantly promoted growth and lowered diarrhea rate in weaned piglets. Furthermore, FHF enhanced intestinal morphology and barrier function, and modulated gut microbial composition by increasing the relative abundance of *Limosilactobacillus*, *Lactobacillus*, and *Phascolarctobacterium*. This modulation was accompanied by increased short-chain fatty acid production, especially butyrate and acetate. By improving intestinal structure, attenuating inflammatory responses, and promoting beneficial microbiota-derived metabolites, FHF may support intestinal function and pig growth after weaning.

## Figures and Tables

**Table 1:** The chemical composition of FHF.

Ingredient	Content (%) in the Total Weight
Water content	3.26 ^1^
pH	3.82
Dry matter	96.70
Crude protein	13.03
Crude fat	1.64
Crude fiber	16.80
Crude ash	8.17

^1^ Due to rounding, water content and dry matter may not sum exactly to 100%.

**Table 2:** Effects of FHF supplementation on intestinal structure in weaned piglets.

Location	Item	Control	A1	A2	*p*-Value
Duodenum (μm)	VH	471.1 ± 10.3 ^b^	530.3 ± 12.4 ^a^	543.6 ± 12.9 ^a^	<0.001
	CD	246.3 ± 8.2	243.5 ± 9.6	243.7 ± 8.4	0.968
	VH/CD	1.9 ± 0.04 ^b^	2.2 ± 0.06 ^a^	2.3 ± 0.02 ^a^	<0.001
Jejunum (μm)	VH	508.9 ± 13.4	530.6 ± 21.9	549.5 ± 23.8	0.391
	CD	243.1 ± 4.7	233.3 ± 6.0	240.1 ± 10.2	0.752
	VH/CD	2.1 ± 0.03 ^b^	2.3 ± 0.03 ^a^	2.3 ± 0.04 ^a^	0.002
Ileum (μm)	VH	423.6 ± 10.5 ^b^	430.1 ± 8.7 ^ab^	457.9 ± 8.6 ^a^	0.046
	CD	232.9 ± 8.6	236.3 ± 7.5	243.2 ± 7.6	0.671
	VH/CD	1.8 ± 0.04	1.8 ± 0.03	1.9 ± 0.02	0.260

Data are expressed as mean ± SEM (n = 6). VH, villus height; CD, crypt depth; VH/CD, the ratio of villus height to crypt depth. Different superscript letters (a, b) within the same row indicate significant differences (*p* < 0.05).

## Data Availability

The data supporting this study are available through the NCBI BioProject database under accession number PRJNA1432004.

## Supplementary Materials

The following supporting information can be downloaded at: https://www.mdpi.com/article/10.3390/ani16081254/s1, Table S1: Identification of the top 20 chemical constituents in FHF by UHPLC-MS/MS analysis; Table S2: Ingredient and nutrient composition of the basal weaning diet; Table S3: Specific primers of related genes; Figure S1: Superclass distribution of bioactive constituents in FHF; Figure S2: Representative H&E-stained sections of the small intestine; Figure S3: Immunofluorescence analysis of phosphorylated NF-κB p65 (p-p65) expression in the jejunum of weaned piglets. Materials and Methods S1.1: UHPLC–MS/MS analysis of FHF extract; Materials and Methods S1.2: Histological and immunofluorescence analyses; Materials and Methods S1.3: Quantitative real-time PCR analysis; Materials and Methods S1.4: Western blot analysis; Materials and Methods S1.5: GC-MS analysis of SCFAs.
