# Supplementary material for: A Fermented Herbal Formulation Improves Intestinal Health and Growth Performance in Post-Weaning Piglets
Source: Animals (Basel). 2026 Apr 19;16(8):1254. doi: 10.3390/ani16081254 (PMC13114063; doi:10.3390/ani16081254)
Supplement: Supplementary file 1 [file animals-16-01254-s001.zip › animals-4232937-supplementary.pdf]

Supplementary Table S1. Identification of the top 20 chemical constituents in FHF by UHPLC-MS/MS analysis.

**Rank**	**Identification**	**Formula**	**RT (min)**	**m/z**	**Super Class**
1	(E)-5-(1,2,4a,5-tetramethyl-2,3,4,7,8,8a-hexahydronaphthalen-1-yl)-3-methyl-pent-2-enoic acid	C20H32O2	306.0	303.23	Terpenoids
2	(E)-5-(2,3-dimethyl-3-tricyclo[2.2.1.02,6]heptanyl)-2-methyl-pent-2-enoic acid	C15H22O2	282.6	233.15	Terpenoids
3	11-Hydroxy-9,12-octadecadienoic acid	C18H32O3	268	295.23	Lipids
4	Corolic acid	C18H32O3	268	295.23	Lipids
5	13-hydroxy-9,11-octadecadienoic acid	C18H32O3	254.6	295.23	Lipids
6	9,12,13-Trihydroxy-10-octadecenoic acid	C18H34O5	243.6	329.23	Lipids
7	9,10,13-Trihydroxy-11-octadecenoic acid	C18H34O5	243.6	329.23	Lipids
8	Pinellic acid	C18H34O5	227	329.23	Lipids
9	15,16-Dihydroxy-9,12-octadecadienoic acid	C18H32O4	226.7	311.22	Lipids
10	9,10-Dihydroxy-12-octadecenoic acid	C18H34O4	224.2	313.24	Lipids
11	12,13-Dihydroxy-9-octadecenoic acid	C18H34O4	224.2	313.24	Lipids
12	12,13-DHOE	C18H34O4	224.2	313.24	Lipids
13	9,10-DHOE	C18H34O4	224.2	313.24	Lipids
14	1,2,3-trihydroxy-5-oxocyclohexanecarboxylate	C7H10O6	223.3	189.04	Shikimates
15	3,4,5-Trihydroxycyclohex-1-enecarboxylic acid	C7H10O5	223.3	173.05	Shikimates
16	Shikimic acid	C7H10O5	223.3	173.05	Shikimates
17	(E)-4-Hydroxy-3-methoxycinnamic acid	C10H10O4	211.3	193.06	Phenylpropanoids
18	Ferulic acid	C10H10O4	211.3	193.06	Phenylpropanoids
19	Isoferulic acid	C10H10O4	211.3	193.06	Phenylpropanoids
20	2-isopropyl-5-methylphenol	C10H14O	211.1	149.10	Terpenoids

Supplementary Table S2. Ingredient and nutrient composition of the basal weaning diet.

**Items**	**Content**
Ingredients	
Corn	61.20%
Soybean meal	19.30%
Wheat	10.00%
Barley	5.00%
Soybean oil	0.50%
Premix	4.00%
Nutrient Levels	
Digestible Energy (MJ/kg)	13.90
Crude Protein	17.65%
Digestible Lysine	1.14%
Calcium	0.84%
Total Phosphorus	0.63%

Supplementary Table S3. Specific primers of related genes.

**Gene**	**Accession No.**	**Sequences (5'→3')**	**Product size (bp)**
IL-6	NM_214399.1	Forward: CTGGGTTCAATCAGGAGACCT<br>Reverse: TTCCCTTTTGCCTCAGGGTC	165
IL-8	NM_213867.1	Forward: TGGACCCCAAGGAAAAGTGG<br>Reverse: TGTTGTTGCTTCTCAGTTCTCTT	70
IL-1β	NM_214055.1	Forward: CCAATTCAGGGACCCTACCC<br>Reverse: GTTTTGGGTGCAGCACTTCAT	174
TNF-α	NM_214022.1	Forward: GGCCCAAGGACTCAGATCAT<br>Reverse: CTGTCCCTCGGCTTTGACAT	82
Occludin	NM_001163647.2	Forward: CAGGTGCACCCTCCAGATTG<br>Reverse: ATGTCGTTGCTGGGTGCATA	167
Claudin1	NM_001244539.1	Forward: TCTTTCTTATTTCAGGTCTGGCT<br>Reverse: ACTGGGGTCATGGGGTCATA	91
ZO-1	XM_021098856.1	Forward: AGAGGAAGCTGTGGGTAACG<br>Reverse: TCACCGTGTGTTGTTCCCAT	192
GAPDH	NM_001206359.1	Forward: CCTCCCCGTTCGACAGACA<br>Reverse: GATGCGGCCAAATCCGTT	97

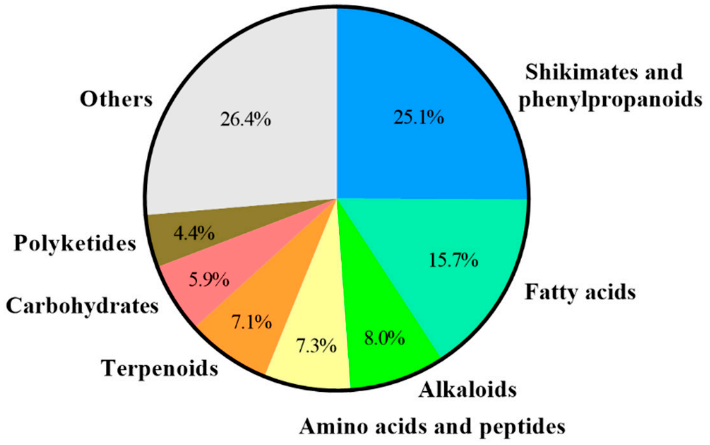

Supplementary Figure S1. Superclass distribution of bioactive constituents in FHF.

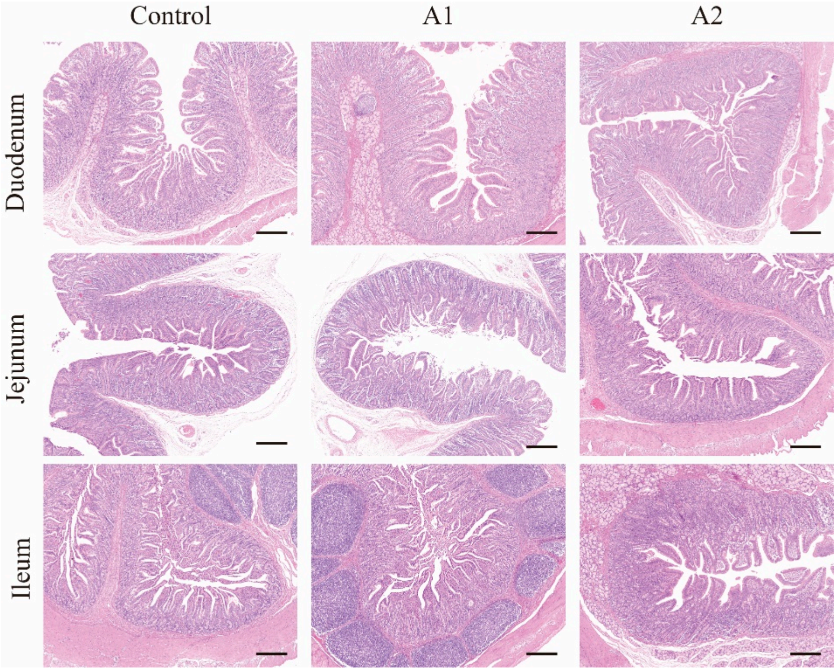

Supplementary Figure S2. Representative H&E-stained sections of the small intestine. Scale bar = 200 μm.

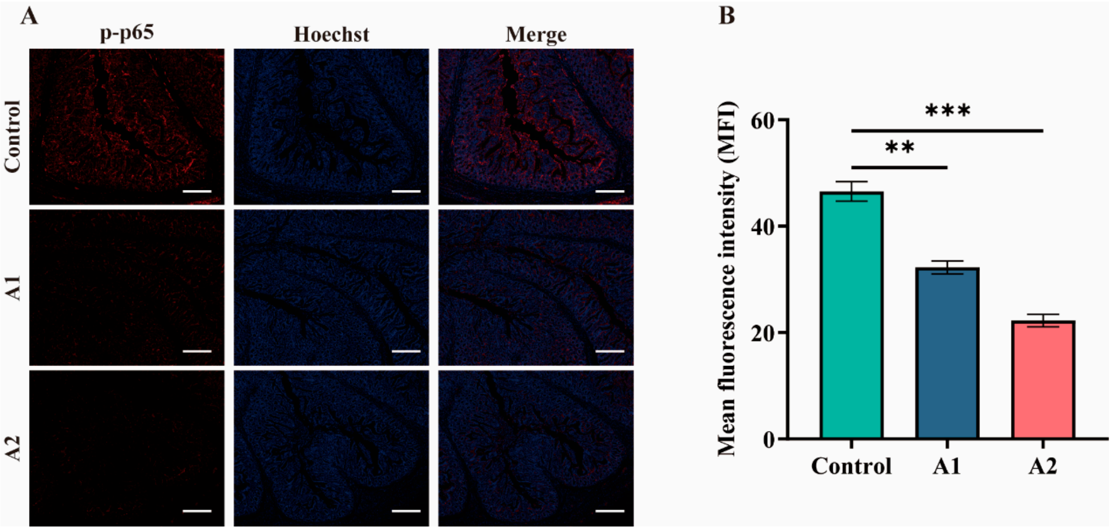

Supplementary Figure S3. Immunofluorescence analysis of phosphorylated NF-κB p65 (p-p65) expression in the jejunum of weaned piglets. Scale bar = 500 μm. (**A**) Representative immunofluorescence images of p-p65 staining in the Control, A1, and A2 groups. (**B**) Quantitative analysis of mean fluorescence intensity (MFI) of p-p65.

Materials and Methods

1.1 UHPLC-MS/MS Analysis of FHF Extract

The chemical profile of the FHF extract was analyzed using a Vanquish UHPLC system coupled with an Orbitrap Exploris 120 mass spectrometer (Thermo Fisher Scientific). Separation was performed on a Phenomenex Kinetex C18 column (50 mm × 2.1 mm, 2.6 μm). The mobile phases consisted of 0.01% acetic acid in water (solvent A) and isopropanol/acetonitrile (1:1, v/v) (solvent B). Data were acquired under electrospray ionization (ESI) in both positive and negative modes using Xcalibur v4.4. The raw data were converted to the mzXML format via ProteoWizard, and peak detection, extraction, and alignment were performed using an XCMS-based R package. Metabolites were identified by matching MS/MS spectra against the BiotreeDB (V3.0) database.

1.2 Histological and Immunofluorescence Analyses

Samples from the duodenum, jejunum, and ileum were fixed in 4% (w/v) para-formaldehyde (Biosharp, Hefei, China). The tissues were then passed through graded ethanol solutions (50–100%), cleared with xylene, and embedded in paraffin. Paraffin-embedded tissues were cut into 5 μm sections with a rotary microtome (Leica Microsystems, Wetzlar, Germany). The sections were then placed on glass slides and dried overnight at 40 °C. Sections were deparaffinized, rehydrated, and stained with hematoxylin and eosin (H&E) for morphological assessment. Images were captured using a Nikon Eclipse CI microscope (Nikon Instruments, Tokyo, Japan). Villus height (VH) and crypt depth (CD) were measured using ImageJ software (NIH, Bethesda, MD, USA). For each tissue section, six intact villi were measured across three consecutive fields of view.

Paraffin-embedded sections were deparaffinized, rehydrated, and subjected to antigen retrieval in citrate buffer (pH 6.0) at 95 °C for 15 min. After cooling to room temperature, sections were blocked with 5% bovine serum albumin (BSA; BioFroxx, Einhausen, Germany) for 30 min. The sections were then incubated overnight at 4 °C with a primary antibody against phospho-NF-κB p65 (Cat. No. 82335-1-RR, Proteintech, Wuhan, China). After washing with PBS, sections were incubated with a fluorophore-conjugated Goat anti-Rabbit IgG (H+L) secondary antibody (ABclonal, Wuhan, China) for 1 h at room temperature in the dark. Nuclei were counterstained with DAPI. Slides were mounted with anti-fade mounting medium and examined using a fluorescence microscope (Olympus, Tokyo, Japan). Fluorescence intensity was quantified as mean fluorescence intensity (MFI) within defined regions of interest using ImageJ software.

1.3 Quantitative Real-Time PCR Analysis

Total RNA was extracted from IPEC-J2 cells and jejunal tissue samples using an RNA extraction kit (ComWin Biotech, Beijing, China) according to the manufacturer's instructions. The concentration and purity of RNA were determined using a NanoDrop ND-2000 spectrophotometer (Thermo Fisher Scientific, Wilmington, DE, USA). One microgram of total RNA from each sample was reverse-transcribed into complementary DNA (cDNA) using a reverse transcription kit (Accurate Biology, Changsha, China). Quantitative real-time PCR was performed using a commercial qRT-PCR kit (Accurate Biology, Changsha, China) on a CFX96™ Real-Time PCR Detection System (Bio-Rad Laboratories, Hercules, CA, USA). All samples were analyzed in triplicate.

The thermal cycling conditions were as follows: initial denaturation at 95 °C for 5 min,

followed by 40 cycles of denaturation at 95 °C for 5 s and annealing/extension at 60 °C for 15 s. A melting-curve analysis was performed to verify amplification specificity. Glyceraldehyde-3-phosphate dehydrogenase (GAPDH) served as the internal reference gene. Primer sequences for target and reference genes are listed in Supplementary Table 3, and all primers were synthesized by Sangon Biotech (Shanghai, China). Relative gene expression levels were calculated using the $2^{-\Delta\Delta Ct}$ method after confirming comparable amplification efficiencies for all primer pairs.

1.4 Western Blot Analysis

Total protein was extracted from jejunal tissues. Tissues were lysed using RIPA lysis buffer (Pumoke Biotechnology, Wuhan, China) supplemented with 1% protease inhibitor cocktail and 1% phosphatase inhibitor cocktail (ComWin Biotech, Beijing, China). Protein concentrations were determined using a BCA assay kit (Thermo Fisher Scientific, Waltham, MA, USA). Equal amounts of protein (30 μg per lane) were mixed with 5× loading buffer, denatured at 95 °C for 5 min, and separated by 10% SDS–PAGE, followed by electrotransfer onto PVDF membranes (Millipore, Billerica, MA, USA).

The membranes were blocked with TBST containing 5% (w/v) skim milk (BD Biosciences, Sparks, MD, USA) for 1 h at room temperature and incubated overnight at 4 °C with primary antibodies against Occludin (Proteintech, 27260-1-AP), Claudin1 (Proteintech, 28674-1-AP), ZO-1 (Proteintech, 21773-1-AP), and β-Actin (Abclonal, AC026). β-Actin was used as the loading control. After washing with TBST, membranes were incubated with HRP-conjugated Goat anti-Rabbit IgG secondary antibody (Abclonal, AS014) for 1 h at room temperature. Protein bands were visualized using an ECL Super Kit (Sparkjade, Qingdao, China) and band intensities were quantified using Image Lab™ software (Bio-Rad Laboratories, Hercules, CA, USA) and normalized to β-Actin.

1.5 GC-MS Analysis of SCFAs

After collection, colonic samples were rapidly frozen in liquid nitrogen and kept at −80 °C before measurement. Acetic, propionic, butyric, isobutyric, valeric, isovaleric, and hexanoic acids were then measured by gas chromatography–mass spectrometry (GC–MS). Approximately 0.5 g of frozen colonic content was mixed with 1 mL distilled water, followed by centrifugation at 12,000 × g for 10 min at 4 °C. Then, 0.2 mL of 25% (w/v) metaphosphoric acid containing 2-ethylbutyric acid as an internal standard was added to the supernatant. After vortexing for 1 min and incubation on ice for 30 min, the mixture was centrifuged again at 12,000 × g for 10 min at 4 °C. The final supernatant was filtered through a 0.22 μm membrane filter and injected into a GC–MS system (Agilent Technologies, Santa Clara, CA, USA) equipped with an HP-FFAP capillary column (30 m × 0.25 mm × 0.25 μm). The oven temperature program was as follows: initial temperature at 90 °C for 1 min, ramped to 120 °C at 10 °C/min, then ramped to 200 °C at 5 °C/min, and held at 200 °C for 5 min. Helium was used as the carrier gas, with a flow rate of 1.0 mL/min. The injector and transfer line temperatures were maintained at 250 °C. SCFA concentrations were determined from calibration curves prepared using analytical-grade standards (Sigma-Aldrich, St. Louis, MO, USA).
